# A Computationally Optimised Structural Integrity Sequence Enhances Vaccine Stability, Yield and Safety Profile

**DOI:** 10.21203/rs.3.rs-7642777/v1

**Published:** 2025-09-30

**Authors:** Arthur Sarron, Sun B. Sowers, Yaroslav Tsybovsky, Heather Colley, Stephen N. Crooke, Guillaume B. E. Stewart-Jones, Ian M. Overton

**Affiliations:** Queen’s University Belfast; Centers for Disease Control and Prevention; Frederick National Laboratory for Cancer Research sponsored by the National Cancer Institute; Centers for Disease Control and Prevention; Centers for Disease Control and Prevention; Mevox Ltd; Queen’s University Belfast

## Abstract

Recombinant vaccines are a cornerstone of global health, exemplified by the eradication of type 3 wild poliovirus in 2020 due to extensive vaccination campaigns. However, sequence similarity between vaccine antigens and human proteins could theoretically risk autoimmune reactions via molecular mimicry. We present the ‘Vaccine Candidate de-Risking and Stabilisation’ (VaCRiSta) computational pipeline; integrating sequence similarity searches, epitope prediction, homology modeling, and molecular dynamics simulations to de-risk and structurally stabilize vaccine candidates. As a case study, we applied VaCRiSta to the GCN4 trimerization domain in the context of the mumps F protein. The optimized sequence (GCN4_QM) eliminates 139 7-mer and 50 8-mer human proteome matches, is predicted to enhance stability of the trimeric F protein assembly and doubles protein expression yield (2.2 vs. 1.1 mg/L) for a mumps vaccine candidate. GCN4_QM maintains structural integrity, confirmed by negative-stain electron microscopy, and elicits approximately 3-fold higher neutralizing antibody titers in mice (*p* < 0.0407). GCN4_QM is a useful structural module for protein engineering and multimeric vaccine design, particularly in replacement of a transmembrane region to ensure the solubility of a trimeric protein. Accordingly, the VaCRiSta approach may support vaccine safety, stability and yield, potentially providing benefits for clinical efficacy and delivery to populations.

## Introduction

Vaccination has transformed global health by preventing the spread of infectious diseases and significantly reducing associated mortality. In 2010, the World Health Organization, together with the Global Immunisation Vision and Strategy programme, recorded a decrease in the mortality due to measles infection by 90% for infants and 78% for the general population respectively when compared to the early 2000s [1]. A reduction in mortality due to infection has followed multiple vaccination campaigns around the world to date. Indeed, prophylactic vaccination has led to the control and elimination of a number of infectious diseases which would otherwise have cost many lives and put intense strain on healthcare systems. Young infants, the immunocompromised and the elderly are particularly at risk of serious complications from infectious disease [2]. Vaccination provides efficient, low cost and long term protection against infection across age groups. More recently, vaccination has been endorsed in the prevention of cervical cancers caused by HPV or hepatitis viruses. Additionally, herd immunity effects and the economic advantages of reduced disease burden, such as lower morbidity, mortality, caregiving costs, and productivity losses, make vaccination one of the most impactful public health tools available [3–5].

Autoimmune reactions can result in morbidity or even death and may be caused by exposure to foreign antigens, including infection [6–10]. Important categories of autoimmune reactions are: i) molecular mimicry; ii) bystander activation of autoreactive cells and iii) activation of polyclonal T-cells with superantigens. Molecular mimicry can arise from similarity between microorganisms and human proteins, resulting in pseudo self-antigens being presented to T lymphocytes [7]. There are numerous overlaps with pentamer and hexamer human peptides in the genomes of infectious organisms, while heptamer and octamer overlaps occur at lower frequency [8, 9]. Accordingly, potential improvements in vaccine technology could be gained from efforts to derisk vaccine compatibility against individual human genomes.

The availability of 3D protein structure is a crucial factor for rational vaccine engineering. The Alphafold2 software marked a step change in protein structure prediction and is having significant impact across biomedical sciences [11, 12]; although further improvements are warranted for example some Alphafold2 predictions include low confidence regions that are represented by filaments in the 3D protein structure [13]. Additional algorithms for structure prediction and design include Rosetta [14] and ProteinMPNN, which performs well in experimental validation [15, 16]. Engineering protein stability is an important consideration in vaccine development, conferring conformational longevity of key neutralization epitopes [17]; for example the Protein Repair One Sto Shop (PROSS) server supports structure-based design of stable and highly expressed proteins [18].

The GCN4 protein is a parallel alpha-helical leucine zipper coiled coil [19] and binds to the promoter of biosynthetic genes, for example in yeast. GCN4-mediated stabilisation of viral protein trimers has been shown to be a fruitful strategy in engineering self-assembling trimeric antigens for vaccine development [20, 21, 22]. The paramyxovirus fusion glycoprotein functions to bring together the viral and host cell membranes, permitting entry of the viral genome into the host cell. Paramyxoviruses include medically important viruses such as parainfluenza, measles, mumps and nipah virus [23]. Crystallisation of the prefusion conformation of the parainfluenza 5 F glycoprotein was challenging due to spontaneous adoption of the postfusion conformation when expressed without the transmembrane region [24]. Subsequently, Yin *et al*. stabilised the prefusion parainfluenza 5 F glycoprotein by appending a GCN4 coiled-coil region to mimic the transmembrane region providing soluble protein production and resulting in a crystal structure [25].

In this paper, we demonstrate a novel computational vaccine engineering workflow, applied to GCN4-stabilised trimerisation as a case study. New designs, including semi-automated sequence-based selection of amino acid alterations, are monitored with homology modelling and molecular dynamics simulations. The resultant coiled-coil trimerisation region (GCN4_QM) has reduced sequence similarity with the reference human proteome, enhanced stability, an enhanced bioinformatic sequence safety profile and a 2-fold increase in expression yield. We evaluated the GCN4_QM Structural Integrity Sequence (SIS) in the context of a mumps prefusion F/HN (Pre-F/HN) chimeric antigen [22], and observed equivalent size-exclusion chromatography and negative-stain electron microscopy properties. The mumps pre-F/HN GCN4_QM improved immunogenicity in mice, compared with the pre-F/HN immunogen stabilized with the parental GCN4 sequence.

## Methods

**Figure 1:**
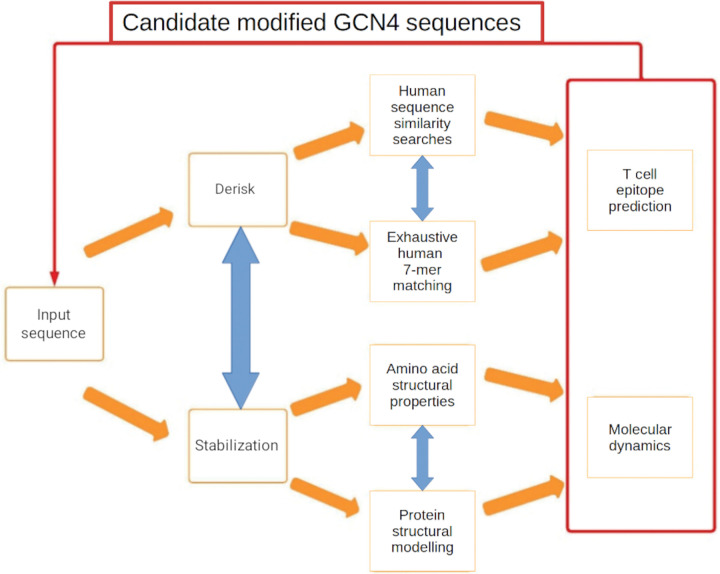
Candidate vaccine derisking and stabilisation pipeline. The input protein sequence is analysed in two complementary, integrated streams; derisking and stabilization. The derisking phase removes sequence similarity between the input sequence and the human proteome, including T-cell epitope screening. The stabilisation step seeks to conserve the physicochemical properties of amino acid residues, to preserve protein structure and to enhance structural stability. Molecular dynamics simulations are the final step in assessing the stability of candidate protein modifications. In practice, candidate GCN4 sequence modifications resulted in new similarities with human protein sequences that were not returned by earlier searches. Accumulated sequence matches are considered together as part of the sequence optimisation process.

Optimisation of the candidate protein sequence was carried out in two linked streams; derisking and stabilization (Fig. 1). Derisking involved sequence searching against the human proteome [15] to identify modifications that may reduce similarity. Stabilization applied structural modelling, seeking to improve or maintain protein stability; including screening for potential introduction of protease cleavage sites. The final stage of the derisking and stabilization processes were respectively screening against T-cell epitopes and molecular dynamics simulations. Results from both streams were integrated to inform sequence modifications and the process was applied iteratively.

### Sequence-based derisking

Comparisons to the human proteome were carried out with a) BLASTP, for sequence matching [26] b) bespoke computer code for exhaustive k-mer (peptide) searching and c) PSI-BLAST to explore alignments across greater evolutionary distance [26]. Sequence modifications and alignment against the human proteome were carried out for assessment and elimination of similarities to human proteins that might be introduced into the modified GCN4 sequences. The iterative modification process also considered elimination of potential autologous T-cell epitopes.

#### Investigating trimerisation region sequence matches to human proteins

**Figure 2:**
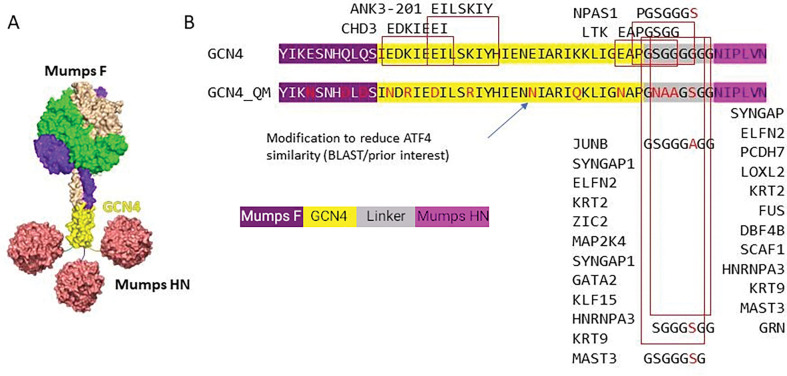
Eliminating matches to the human proteome found in the GCN4 trimerisation region. A) 3D structural model of the Mumps Fusion (F) protein in prefusion conformation, GCN4 trimerisation region (yellow), linker and Mumps hemaglutinin-neuraminidase (HN) protein; representing the candidate mumps vaccine considered in this study. B) The GCN4_init and GCN4_QM sequences are shown with annotated 7-mer and 8-mer matches to the human proteome (red boxes); these matches are eliminated in the GCN4_QM sequence. The text background colour for the sequences corresponds to Mumps F (purple), GCN4 (yellow), linker (grey) and Mumps HN (magenta). Residues that differ to GCN4_init are shown in red and were rationally modified to produce GCN4_QM.

The first derisking step searches the input protein sequence against the human proteome [10]. The current study focussed upon the parental GCN4 trimerisation region (GCN4_init) plus an additional eleven residues either side in the vaccine candidate sequence (Fig. 2, Supplementary Figure S1), in order to evaluate potential cross-reactive epitopes arising from a fusion of GCN4 and juxtaposed viral sequences. Searches with protein BLAST (BLASTP) and PSIBLAST [26] took sensitive threshold parameter values because establishment of a statistically significant evolutionary relationship is not required for potential immune cross-reactivity. Accordingly, the BLASTP expectation value (e-value) threshold was set to 1000 to allow sensitive detection of short sequence matches using default parameters with the human proteome search databases (UniProtKB/Swiss-Prot and Ensembl) [27, 28]. A complementary search strategy deployed PSI-BLAST, run for three iterations with e-value threshold of 10^− 3^ [26] and a final iteration taking e-value ≤ 10. The search database was NCBI NR. Other parameters were word size = 3, BLOSUM62 matrix, gap cost = 11, gap extension = 1. Multiple Sequence Alignment (MSA) was performed with ClustalO and visualised in Jalview [29, 30].

T cells recognise peptides presented at the cell surface by Major Histocompatibility Complex (MHC) proteins. MHC class I typically recognises 8-mers or 9-mers [2, 31]; however ‘mix and match’ dual binding of non-contiguous peptides, including 7-mers, might potentially result in epitope presentation [32]. Importantly, exogenous antigens, including material from vaccination, could be processed *via* MHC class I (‘cross-presentation’) as well as the canonical MHC class II pathway [33, 34].

Genetic variation at the population level influences potential autoimmunity, driving differences in the mechanisms that regulate immune responses to self-antigens. Therefore, vaccine derisking benefits from consideration of potential variation across populations. In order to mitigate potential undesirable epitopes arising from population variation we sought to eliminate all identical or sequence-similar 7-mers from the GCN4 Structural Integrity Sequence (SIS). We reasoned that identical 8-mers or 9-mers, which could be recognised by the MHC class I pathway, are highly unlikely to arise through population variation if any exact 7-mer matches to the reference human proteome are eliminated. We developed computer code (Kmer_finder, https://github.com/overton-group/Kmer_finder) to identify K-mers between a query sequence and the human proteome. The Kmer_finder approach proceeds through the following key steps: initially, contiguous k-mer sequences are identified in the Ensembl human proteome, forming a human k-mer reference database (Human_Kmer_DB). The query protein sequence is split into contiguous k-mers (query_Kmers) and then query_Kmers are compared to Human_Kmer_DB. We took values of k = 7, k = 8; while 7-mers are unlikely to have high affinity with MHC class I or class II peptide binding grooves, these might potentially contribute to 8-mer or 9-mer matches arising from natural variation across the human population. We reasoned that exclusion of the 7-mers represents a conservative threshold for elimination of potential undesirable sequence matches between the query sequence and the human proteome. Accordingly, the 7-mer exclusion threshold may help to mitigate potential hybrid peptide reactivity with the MHC. Visualisation and MSA of matching 7-mers with the GCN4 sequence was produced with ClustalO and JalView [29, 30].

#### T-cell epitope screening

T cells recognise peptides presented at the cell surface by MHC proteins that have a peptide-binding groove, canonically recognising peptides from 8–10 amino acids (9-mers favoured by class I) and 13–25 amino acids (class II) [2, 31]. As noted above cross-presentation may occur where peptides are processed through MHC class I [33, 34], however the majority of autoimmune reactions are thought to involve MHC class II presentation [35, 36, 37]. We predicted T-cell epitopes with TepiTool [38] and NetMHCpan [39], the GCN4-derived query sequences were analysed for matches to the MHC class I (A-G) and II (DP, DQ, and DR). All peptide lengths were included (7–14mers for class I and 15mers for class II). The default prediction method was applied (‘IEDB recommended’) and predicted peptides with percentile rank < 10 were taken forwards.

### Protein stabilization

#### Amino acid structural properties

Predicted solvent accessibility and secondary structure were obtained from JPred [40], protein disorder was predicted by GlobPlot [41] and Kyte-Doolittle hydrophobicity [42] was visualised in JalView [30]. Protease cleavage sites were predicted using EMBOSS tools [43].

#### Homology modelling

Discovery Studio Visualizer was used for the structure visualization [44]. The query protein sequence and its modified versions were input for the homology modelling software MODELLER 10.2 [45] with the template structure (1GCM) obtained from the PDB [46, 47]. Five models were proposed per query sequence and the global energy free energy determined. The 3D models with lowest normalized Discrete Optimized Protein Energy (DOPE) scores were taken as input into molecular dynamic simulations. Helical wheel representations were calculated with the EMBOSS ‘pepwheel’ software [43] and residue interactions were determined by LigPlot [48].

#### Molecular dynamics

Molecular dynamics simulations were performed using GROMACS 2020.4 [52]. An AMBER03 force field was applied to the proteins [53], which were placed into a cubic box and filled with water as solvent. The size of the box is set to the diameter of the system and centred with a separation of 1.0 nm between the protein and the edge of the box. To neutralize the system, chloride and sodium atoms replaced pseudo-randomly selected water molecules. The system was energy minimized using a steepest descent minimization with an initial step size of 0.1 nm, maximum number of steps of 50000 and a maximum force of 1000 kJmol^− 1^nm^− 1^. According to the NVT ensemble, the system temperature was equilibrated at 300 K for 100 ps and then the pressure was equilibrated to 1 bar for 100 ps using Berendsen coupling [54]. The protein position was restrained during the equilibration of temperature and pressure. Each molecular dynamics simulation was run for 50 ns.

### Protein expression

Pre-F/HN glycoproteins were expressed by transfection in 293-Expi cells (Thermo Fisher) using Lipofectamine transfection reagent (Thermo Fisher) according to the manufacturer’s protocol. Transfected cells were incubated in shaker incubators at 120 rpm, 37°C, 8% CO_2_ overnight. On the second day, one tenth culture volume of CellBooster medium (ABI scientific) was added to each flask of transfected cells and cell cultures were incubated at 120 rpm, 37°C, 8% CO_2_ for an additional 4 days. 5 days post-transfection, cell culture supernatants were harvested and proteins were purified from the supernatants using tandem Ni2+ (Roche) and Streptactin (IBA) affinity purification. The C-terminal purification tags were removed by thrombin digestion at room temperature overnight and proteins were further purified by SEC in a Superdex 200 column (GE) in PBS.

### Negative-Stain Electron Microscopy

Pre-F/HN proteins were purified to > 95% purity were diluted with 10 mM HEPES, pH 7.0, 150 mM NaCl, adsorbed to a freshly glow-discharged carbon film-covered grid, washed with the same buffer, and stained with 0.7% uranyl formate. Images were collected at a magnification of 100,000 using SerialEM (55) on an FEI Tecnai T20 microscope equipped with a 2k × 2k Eagle CCD camera and operated at 200 kV. The pixel size was 0.22 nm. 2D classification was performed in Relion 3 [56].

### Characterizing the Immunogenicity of Pre-F/HN designs in mice

To assess the immunogenicity of recombinant mumps Pre-F-HN designs with either GCN4_init or GCN4_QM measured by elicitation of neutralizing antibodies, groups of 8 female CB6F1/J mice were immunized twice, at weeks 0 and 3 with 10 μg of recombinant mumps F glycoprotein trimer designs combined with 10 μl Alhydrogel (CRODA) and 2 weeks after immunization, sera were assessed for heterologous mumps neutralization *in vitro.* Neutralizing antibody titers were determined using a plaque reduction neutralization (PRNT) assay. Neutralizing antibody titers to the genotype G mumps virus were determined by the PRNT assay as described previously [57]. Sera were heat-inactivated for 30 min and serially diluted 2-fold starting from 1:2 to 1:2,048 and mixed with an equal volume of mumps virus that yielded approximately 40 to 60 plaque-forming units, containing a final dilution ranging from 1:4 to 1:4,096. PRN ID60 titers were determined to be the highest dilution of serum that gave 60% or higher plaque reduction compared with the average number of plaques formed in the absence of serum or monoclonal antibody by using the Kärber formula.

## Results and Discussion

### Sequence similarity between the GCN4 trimerisation region and the human proteome

In order to investigate matches between the parental GCN4 trimerisation region (GCN4_init) and the human proteome, we developed the ‘K-mer_finder’ software to identify identical (matching) contiguous sequence regions. Sensitive searches were also carried out with BLASTP, PSIBLAST [26], in order to identify sequence similar regions regardless of the statistical or evolutionary significance of the match. An exhaustive comparison across all 7-mers with Kmer_finder revealed identical matches for GCN4 with 73 unique Ensembl protein identifiers (Supplementary Table S1), these were mostly in the low-complexity linker region and representative examples are shown in Fig. 2. Human homologues of the GCN4_init sequence were identified in PSIBLAST searches; as expected, contiguous regions of identical or very similar amino acids are not typically identified in the more evolutionarily distant matches. The PSIBLAST results identified a low-complexity region of eight amino acids where seven of eight identical residues occurred within the JUNB sequence (Fig. 2, Supplementary Figure S2). We also found T cell epitopes that covered the entire GCN4_init sequence, Table 1 shows the top 40 MHC class I peptides.

Sequence polymorphism could potentially lead to lengthening of the existing regions of similarity between GCN4_init and the human reference proteome. For example, the predicted epitopes ‘KIEEILSKI’ and ‘EILSKIYHI’ (respective predicted IC50 0.4μM, 0.1μM) might cross-react with the membrane-associated protein ANK3 sequence positions 2511–19 (KEILSKIYK; part of the UniProt canonical ANK3 sequence). Individuals with an ANK3 mutation K2511->E would have an 8-mer peptide that matches exactly to GCN4_init and a K2519->H mutation would generate an 8-mer exact peptide match to GCN4_init that falls within the above predicted epitopes. The K2511->E mutation involves just a single nucleotide change (A->G), whereas K2519->H would require two nucleotide changes (e.g. A->C at the 1st and 3rd codon positions). Indeed, the peptide (XEILSKIYX) has favourable auxiliary anchor residues at position four (leucine) and seven (isoleucine), while the negatively charged glutamate at position one (produced by K2511->E) could be a potent primary anchor [39]. Accordingly, we identified possible MHC class I epitopes in GCN4_init with a length of 8 amino acids. Exogenous (extracellular) material, including from vaccination, may be processed by the MHC class I pathway through cross-presentation [33, 34]. Analysis with NetMHCpan4.1 [39] predicted binding for the above sequences to MHC allele HLA-B*18:01, which is present at relatively high frequency in some populations (Supplementary Figures S3 and S4).

### Optimisation of the GCN4 trimerization sequence to enhance safety profile and increase vaccine stability

**Figure 3:**
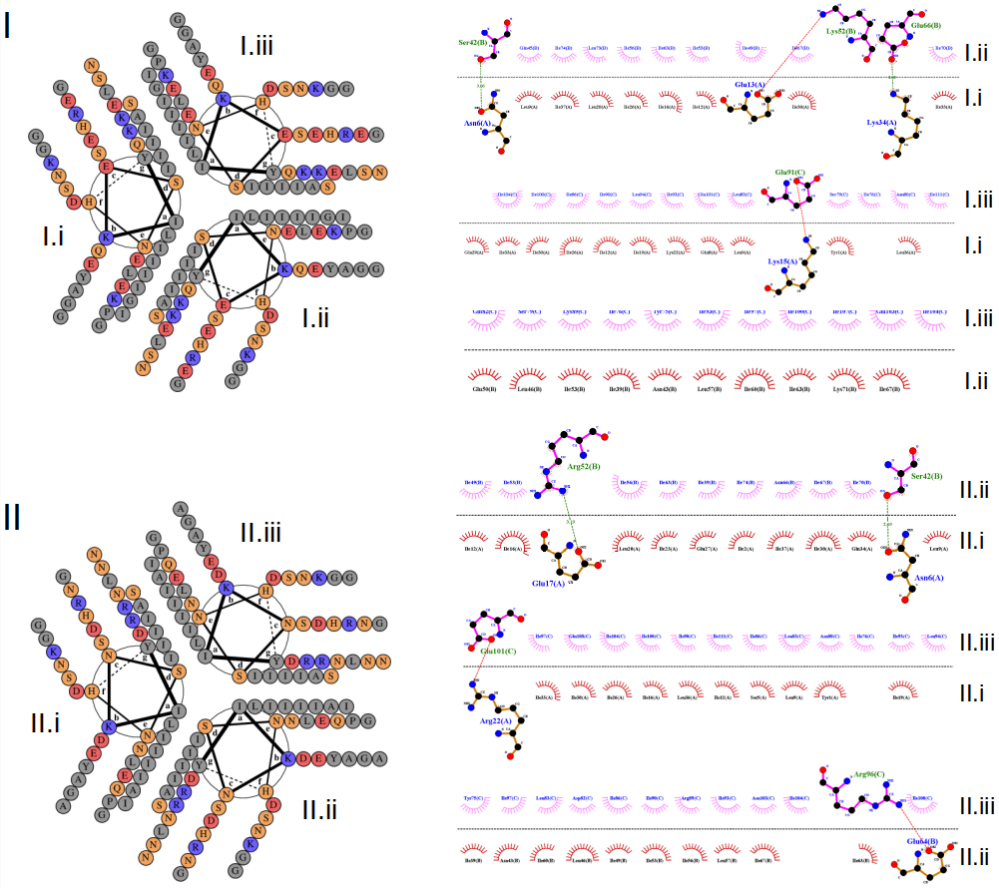
Mapping residue contacts for the GCN4 helices and modification for enhanced stability. Helical wheel representations (left) and LigPlot results (right) are shown for the GCN4_init (I) and GCN4_QM (II) 3D models. Individual helices are labeled (I.i, I.ii, I.iii; II.i, II.ii II.iii). The inner Tyrosine (Y) is the N-terminus for all helices (left). Letter colour shows key biophysical properties: positive charge (blue), negative charge (red), hydrophobic (grey), and polar/other (orange). While the helical wheel representations have rotational symmetry, the interactions between helices in the 3D structural models are not symmetrical, which is reflected in the LigPlot results. Indeed, LigPlot identified Van der Waals/hydrophobic packing, electrostatic interactions (Lys-Glu, red lines) and/or hydrogen bonds (green lines) between GCN4_init helix pairs I.i-I.iii and I.i-I.ii. LigPlot shows GCN4_QM salt bridge hydrogen bonds (green) or electrostatic (red) interactions between all three pairs of helices while only has Van der Waals interactions are found between GCN4_init helices I.ii-I.iii; these differences are expected to result in greater thermodynamic stability for the GCN4_QM trimer.

Rational amino acid modifications were investigated in order to both remove sequence similarity and to improve stability for the GCN4 Structural Integrity Sequence (SIS). MSAs of the GCN4-derived sequences and human candidate matching regions were considered with predicted solvent accessibility, secondary structure and Kyte-Doolitte hydrophobicity [42]. The free energy, radius of gyration and 2D projection of trajectory were taken as indicators of stability. Alteration of the hydrophobic core residues was avoided to help preserve the trimeric hydrophobic packing. To promote stability, we sought to: introduce helix-perpendicular and between-monomer salt bridges, enhance solubility, avoid charge repulsion between structurally proximal side chains, and balance overall charge. Protease sites were also screened in order to prevent cleavage of the engineered protein. Helical wheel representations and LigPlot [48] results for GCN4_init, GCN4_QM are shown in Fig. 3. The inter-helix contacts for GCN4_init and GCN4_QM involve hydrophobic Van der Waals interactions, and electrostatic interactions. Electrostatic interactions for GCN4_init occur between Lysine and Glutamate; however these are replaced by Arginine-Glutamate interactions in GCN4_QM. Arginine binds to negatively charged amino acids more strongly than Lysine with a difference in ΔG of at least – 8.6 kJ/mol for interaction with Glutamate [49]. Additionally, Arginine has a larger positive surface area for electrostatic interactions and is more frequently represented in thermophiles [50, 51]. The pairs of helices II.ii, II.iii (GCN4_QM) and I.ii, I.iii (GCN4_init) have the same number of residues participating in Van der Waals interactions for hydrophobic packing. However, there is an additional Arg-Glu electrostatic interaction for II.ii, II.iii that confers at least – 40 kJ/mol greater thermodynamic stability to the interaction between the II.ii, II.iii pair compared with GCN4_init helices I.ii, I.iii. On the other hand, GCN4_init has interactions between two Lys-Glu pairs within helices I.i, I.ii whereas GCN4_QM helices II.i, II.ii have a single Arg-Glu pair interacting through a salt bridge, as well as hydrogen bonding between Ser-Asn. The Glu-Lys electrostatic interaction between GCN4_init helices I.i, I.ii increases overall thermodynamic stability. However, at physiological temperatures, these Glu-Lys interactions may have limited effect upon the stability of the three-helix bundle in comparison to the interactions that occur between GCN4_QM helices II.i, II.ii. Indeed, all three GCN4_QM helix pairs have electrostatic interactions, which is expected to be important in enhancing the overall stability of the three-helix bundle relative to GCN4_init where one of the three helix pairs has neither hydrogen bonding nor electrostatic interactions (Fig. 3). We also note that the homology model for GCN4_QM appears more stable than for GCN4_init, due to a lower Z-DOPE score (Table 2) [45].

**Figure 4:**
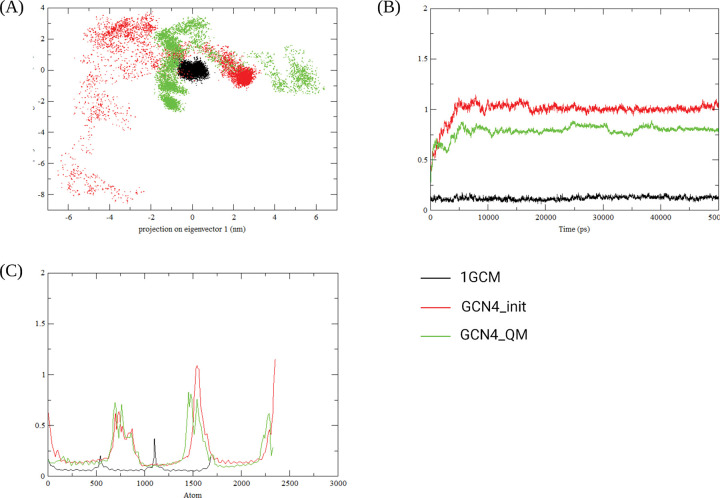
GCN4_QM has greater conformational stability than GCN4_init. (A) 2D projection of Molecular Dynamics trajectories. Each point represents a snapshot of the protein structure, 2D spatial proximity correlates with temporal proximity. GCN4_init (red) adopts a greater range of different conformations than GCN4_QM (green). The yeast protein (1GCM, black) has a tight cluster of conformational states that, on average, are more similar to the GCM4_QM states relative to GCN4_init. (B) GCN4_QM has greater structural similarity to the ‘native’ 1GCM protein than GCN4_init. Root Mean Squared Deviation (RMSD, Y-axis) values relative to the 1GCM reference positions are shown for GCN4_init (red), GCN4_QM (green) and 1GCM (black). RMSD for both GCN4_init and GCN4_QM increases before reaching a plateau around 5 ns. Accordingly, GCN4_QM and GCM1 have greater structural similarity than GCN4_init and GCM1. (C) The GCN4_init sequence shows greater mobility than GCN4_QM. Root Mean Squared Fluctuation (RMSF) shows the time-averaged deviation of atoms across the protein sequence (referenced to t=0 positions), the atom numbering starts at the N-terminus. GCN4_init has high RMSF values that are greater than the GCN4_QM RMSF including at approximate atom positions 1550–1600 and 2300–2400, which include residues Tyr103 and Gly146–Ile153.

We further investigated structural stability using molecular dynamics simulations. The 2D trajectory, Root Mean Squared Deviation (RMSD) and Root Mean Squared Fluctuation (RMSF) are shown in Fig. 4. The GCN4_init model has a greater diversity of conformational states than GCN4_QM with both models reaching a cluster of states located to the right side of their respective trajectories by the end of the simulation (Fig. 4B). GCN4_QM has lower RMSD with the 1GCM reference model and the RMSF identifies three distinct regions where the atoms deviate from their original positions (600–1000, 1300–1750, and around 2250). The GCN4_QM model also has smaller RMSF than GCN4_init. Interactions between R22-E101, R96-E64, and R52-E17 form a stabilising ‘ring’ in the GCN4_QM structure. In comparison, GCN4_init has two interactions in the ‘unstable’ region (K15–E101, and N6–S42). The wild-type yeast GCN4 template structure (PDB identifier: 1GCM) remained compact, in one cluster of states. The RMSD of both GCN4_QM and GCN4_init increases from 0 to 10000 ps followed by a plateau. However, GCN4_QM is more stable than GCN4_init, with broadly lower RMSD. Three protein regions have substantial deviation from their original positions (around atom positions 600–1000, 1300–1750, 2200–2400; Fig. 4C).

**Figure 5:**
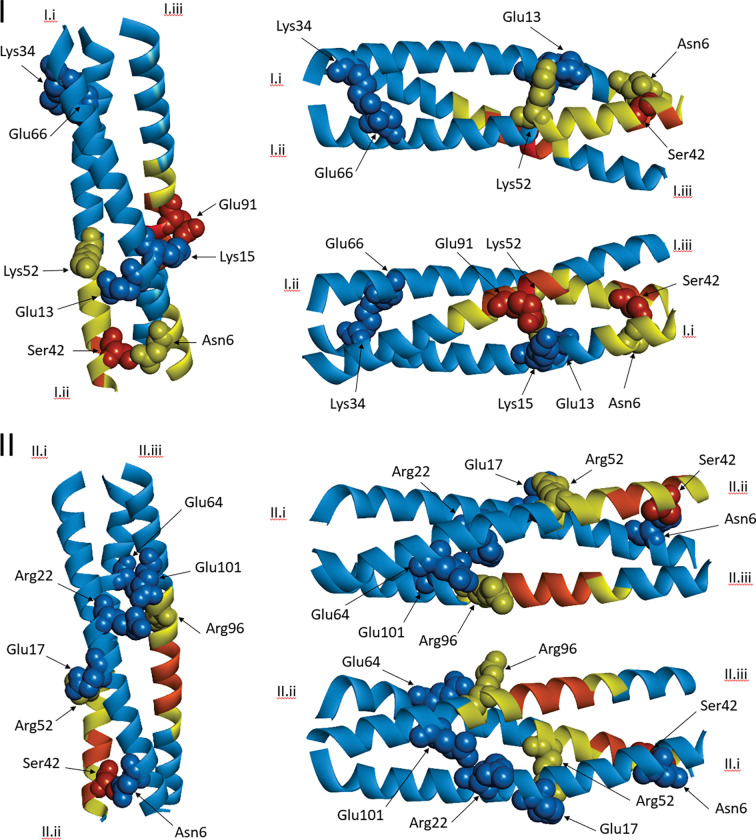
Visualisation of RMS fluctuation for GCN4_init and GCN4_QM homology models. Colour indicates the magnitude of fluctuation: blue (0–0.25Å), yellow (0.25–0.5Å), orange (0.5–1Å), red (>1Å). Van der Waals radii are shown for residues that have inter-helix electrostatic interactions or hydrogen bonding, also labelled with amino acid identity and sequence position. Regions that align with the 1GCM template structure (111 amino acids) are shown. Overall, GCN4_init (I, top) has larger structural fluctuations than GCN4_QM (II, bottom) at the N-terminus of helix i (yellow for GCN4_init, blue for GCN4_QM). Also, the mid-region of helix iii had greater instability for GCN4_init (red) than GCN4_QM (orange). GCN4_init Glu91 on helix I.iii has substantial fluctuations (red) which may disrupt electrostatic interaction with Lys15 (blue) on helix I.i. Large fluctuations for GCN4_init also occur for helix I.ii Ser42 (red), impacting hydrogen bonding with Asn6 (yellow). In contrast, we found low fluctuations for the GCN4_QM residues participating in the electrostatic interaction between helices II.iii (Glu101) and II.i (Arg22); Asn6 is also more stable in GCN4_QM. These results provide a basis to explain stronger inter-helix interactions and therefore predict greater stability of the GCN4_QM three helix bundle.

Three key inter-helix interactions in GCN4_QM form a pseudo-ring in the structure, helping to confer stability; specifically, these residue pairs are Arg22-Glu101, Arg96-Glu64 and Arg52-Glu17 (Figs. 3 and 5, Supplementary Figure S5). Residues involved in key inter-helix interactions for the GCN4_QM model are in structurally stable regions (RMS fluctuation < 0.25Å), shown as blue regions in Fig. 5 panel II; or have intermediate stability (yellow regions, 0.25Å< RMS fluctuation < 0.5Å). Relative to GCN4_QM, larger conformational fluctuations occur for GCN4_init residues participating in key inter-helix interactions (Lys15-Glu91 and Asn6-Ser42); for example, Glu91 is centrally placed in the helix bundle and has RMS fluctuation > 1Å in the GCN4_init model (Fig. 5, panel I).

### Optimisation of the GCN4 sequence doubled candidate vaccine yield

**Figure 6:**
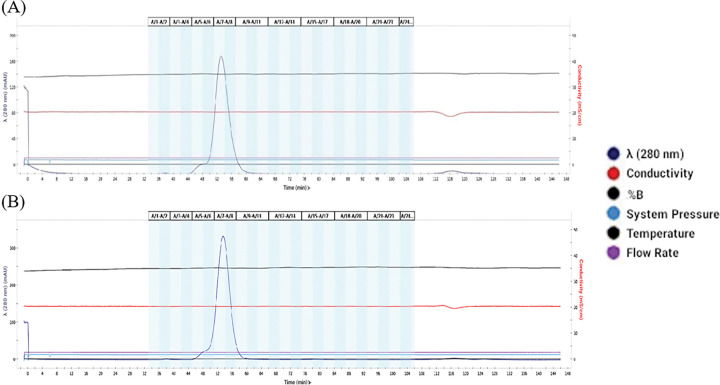
The engineered GCN4_QM trimerisation region produced a doubling in Mumps F-HN candidate vaccine yield. The elution profiles of Mumps F-HN with the GCN4_init (A) and GCN4_QM(B) are shown. The x-axis indicates elution time. The left y-axis shows ultraviolet absorbance at 280 nanometers, representing protein concentration; dark blue line with a peak around 53 minutes. Other parameters include flow rate (purple), temperature (black), system pressure(light blue) and conductivity (red line; y-axis, right side). The GCN4_QM peak is sharper and has a larger area than the GCN4_init peak. Overall yields were respectively 0.11 mg/100ml, 0.22 mg/100ml for GCN4_init, GCN4_QM; accordingly GCN4_QM produced a doubling in yield relative to GCN4_init.

Production of the Mumps fusion (F) in combination with the hemaglutinin-neuraminidase (HN) protein was investigated where stabilisation of the F trimer was achieved by either GCN4_QM or GCN4_init; all other sequence features remained unchanged. Consistent with the predicted increased thermodynamic stability, inclusion of GCN4_QM resulted in a doubling of yield relative to GCN4_init (0.22mg/100ml vs 0.11 mg/100ml; Fig. 6).

### Structural characterization and immunogenic comparison of Pre-F/HN-GCN4_init and Pre-F/HN-GCN4_QM protein vaccine designs

**Figure 7:**
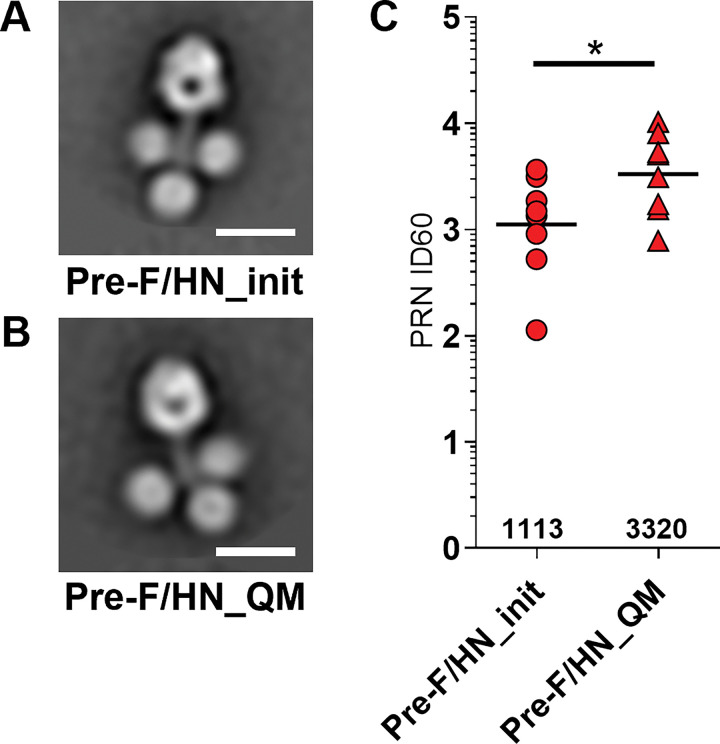
Negative-stain electron microscopy and neutralization titers of vaccines in mice. Representative 2D class averages from negative-stain electron microscopy for the mumps Pre-F/HN protein with the GCN4_init (A) and GCN4_QM (B) are shown. The white scale bar is 10 nm in length. (C) Immunogenicity of the mumps Pre-F/HN protein with the GCN4_init (red circles) or GCN4_QM (red triangles) trimerization designs in CB6F1/J mice after two intramuscular doses at 10ug with Alhydrogel adjuvant at times 0,3 weeks, followed by blood sampling at week 5 for evaluation by plaque reduction neutralization (PRNT) assay with mumps genotype G virus. Geometric mean neutralization titers are shown in each column; mean values are 1634, 4496 with difference between means of 2862 (two-tailed *p*<0.0407, indicated by asterisk; 95% CI 5585, 139; n=8 per group).

Purified mumps Pre-F/HN-GCN4_init and Pre-F/HN-GCN4_QM chimeric antigens were evaluated using negative-stain electron microscopy. Overall, similar structural conformations were observed, showing a prefusion-stabilized trimeric headgroup linked flexibly to three HN head domains (Figs. 7A and 7B). These results confirmed the overall structural architecture of the parental and engineered antigens are broadly identical. CB6F1 mice (n = 8 per group) were immunized with purified Pre-F/HN_init and Pre-F/HN_QM chimeric antigens combined with Alhydrogel adjuvant at weeks 0 and 3 and sera obtained from week 5. To evaluate the elicitation of functional antibodies, we measured the genotype G PRNT from sera collected at week 5, each sample representing a different mouse. Neutralizing antibody titers were observed with the Pre-F/HN_init group (geometric ID_60_ of 1113) that were similar to those observed in [22], however the Pre-F/HN_QM group displayed titers approximately 3.0-fold higher (geometric ID_60_ of 3320) and was statistically significant; comparing GCN4_init (A) and GCN4_QM post two doses (unpaired *t* test, two-tailed *P* <0.0407).

## Conclusions

We present the Vaccine Candidate de-Risking and Stabilisation (VaCRiSta) protocol, applied to the GCN4 vaccine stabilisation region as a case study to produce the GCN4_QM sequence. This engineering pipeline eliminated 139 7-mer matches and 50 8-mer matches to the Ensembl human proteome that had occurred in the GCN4_init sequence. Simultaneously, we designed stronger and new interactions in the GCN4_QM three-helix bundle, providing a structural basis for greater protein stability. Indeed, GCN4_init had neither electrostatic interactions nor hydrogen bonds between chains ii and iii. Results from homology modeling and molecular dynamics demonstrated the expected higher thermodynamic stability for GCN4_QM, which was validated in protein production where a doubling of candidate vaccine yield was achieved. Negative-stain electron microscopy confirmed structural equivalence between the two Pre-F/HN designs and immunization in mice showed a higher level of neutralization was elicited from the Pre-F/HN candidate vaccine that included GCN4_QM. This latter result may have arisen from the additional stability of the GCN4_QM moiety *in vivo*. The engineered GCN4_QM protein is a useful building block supporting the design of engineered soluble trimeric proteins in vaccine development, including for the *Paramyxoviridae* family. The strategy demonstrated here may be widely useful in derisking, for example with further development to consider genetic variation across populations, and to enhance vaccine yield. Safety, stability and yield are critical factors in vaccine development, directly impacting clinical outcomes and cost [59, 60]. Accordingly, VaCRiSta and GCN4_QM are useful in the development of effective vaccines to support population health.

## Supplementary Material

Supplementary Files

This is a list of supplementary files associated with this preprint. Click to download.

• SupplementaryInformation.pdf

## Figures and Tables

**Table 1:** High-scoring predicted T cell epitopes for GCN4_init. The forty top-ranked MHC class I epitopes are shown for analysis of GCN4_init with TepiTool[34]. Ranking is based on predicted IC50 value. Columns showing the start and end positions correspond to the GCN4_init sequence. The percentile rank column identifies the peptide's predicted binding affinity against that of a large set of similarly sized peptides that were randomly selected from the SWISS-PROT database; lower values indicate stronger predicted binding.

Allele	Start position	End position	length	peptide	Predicted IC50 (nM)	Percentile rank
HLA-A*30:01	32	40	9	RIKKLIGEA	29.09	0.13
HLA-A*32:01	22	30	9	KIYHIENEI	42.07	0.05
HLA-B*40:01	12	20	9	IEDKIEEIL	49.81	0.1
HLA-C*03:03	1	9	9	YIKESNHQL	98.45	0.2
HLA-C*03:04	1	9	9	YIKESNHQL	98.45	0.2
HLA-A*68:02	18	26	9	EILSKIYHI	105.94	0.52
HLA-B*18:01	16	24	9	IEEILSKIY	140.42	0.1
HLA-C*12:03	1	9	9	YIKESNHQL	143.71	0.28
HLA-A*02:06	1	9	9	YIKESNHQL	166.93	1.3
HLA-C*03:02	1	9	9	YIKESNHQL	174.73	0.44
HLA-C*16:01	1	9	9	YIKESNHQL	178.13	0.48
HLA-A*68:02	29	37	9	EIARIKKLI	251.91	0.9
HLA-C*17:01	1	9	9	YIKESNHQL	265.88	0.15
HLA-C*12:02	1	9	9	YIKESNHQL	275.4	0.21
HLA-C*02:02	1	9	9	YIKESNHQL	304.75	0.12
HLA-C*02:09	1	9	9	YIKESNHQL	304.75	0.12
HLA-B*08:01	1	9	9	YIKESNHQL	306.6	0.33
HLA-A*02:01	1	9	9	YIKESNHQL	309.14	1.7
HLA-B*40:02	28	36	9	NEIARIKKL	313.75	0.51
HLA-C*14:02	1	9	9	YIKESNHQL	339.51	0.73
HLA-A*68:02	4	12	9	ESNHQLQSI	351.02	1.2
HLA-A*02:06	22	30	9	KIYHIENEI	365.86	2.1
HLA-A*02:06	15	23	9	KIEEILSKI	442.78	2.4
HLA-A*02:01	22	30	9	KIYHIENEI	447.09	2.1
HLA-B*44:02	28	36	9	NEIARIKKL	449.67	0.25
HLA-A*68:02	1	9	9	YIKESNHQL	552.51	1.6
HLA-B*44:03	28	36	9	NEIARIKKL	564.81	0.29
HLA-B*44:03	16	24	9	IEEILSKIY	613.61	0.32
HLA-B*18:01	17	25	9	EEILSKIYH	623.62	0.28
HLA-B*44:02	16	24	9	IEEILSKIY	644.93	0.34
HLA-B*15:25	1	9	9	YIKESNHQL	665.99	2.2
HLA-A*02:06	18	26	9	EILSKIYHI	730.67	3.3
HLA-B*08:01	18	26	9	EILSKIYHI	733.35	0.68
HLA-B*40:02	12	20	9	IEDKIEEIL	779.24	0.99
HLA-C*15:02	22	30	9	KIYHIENEI	907.76	0.48
HLA-B*40:01	28	36	9	NEIARIKKL	1018.86	0.68
HLA-B*15:01	1	9	9	YIKESNHQL	1038.08	2.1
HLA-A*02:06	35	43	9	KLIGEAPGS	1046.09	4
HLA-B*18:01	28	36	9	NEIARIKKL	1096.08	0.43

Optimisation of the GCN4 trimerization sequence to enhance safety profile and increase vaccine stability

**Table 2:** Normalised Z-DOPE score for the GCN4 homology models. GCN4_QM has the lowest score, which suggests greater thermodynamic stability.

Model	Trimer Z-DOPE score
GCN4_QM	−2.98
GCN4_init	−2.85

## Data Availability

Homology models for GCN4_QM and GCN4_init are available as supplementary data files. Other data associated with this study are available upon reasonable request from the corresponding author.
